# Optimizing Nitrogen and Seed Rate Combination for Improving Grain Yield and Nitrogen Uptake Efficiency in Winter Wheat

**DOI:** 10.3390/plants11131745

**Published:** 2022-06-30

**Authors:** Hemat Mahmood, Jian Cai, Qin Zhou, Xiao Wang, Allan Samo, Mei Huang, Tingbo Dai, Mohammad Shah Jahan, Dong Jiang

**Affiliations:** 1 National Technique Innovation Centre for Regional Wheat Production, Key Laboratory of Crop and Ecophysiology in Southern China, Nanjing Agricultural University, Ministry of Agriculture, Nanjing 210095, China; mahmoodhemat@yahoo.com (H.M.); qinzhou@njau.edu.cn (Q.Z.); xiaowang@njau.edu.cn (X.W.); allansamo@njau.edu.cn (A.S.); huangmei@njau.edu.cn (M.H.); tingbod@njau.edu.cn (T.D.); jiangd@njau.edu.cn (D.J.); 2 Department of Agronomy, Agriculture Faculty, Ghazni University, Ghazni 2301, Afghanistan; 3 Department of Horticulture, Sher-e-Bangla Agricultural University, Dhaka 1207, Bangladesh; shahjahansau@gmail.com

**Keywords:** high yield, nitrogen application, N use efficiency, seed rate, winter wheat

## Abstract

Nitrogen (N) supply and seed rate (SR) are two essential factors that affect the accumulation and partitioning of N and dry matter (DM) and, therefore, grain yield (GY) and N use efficiency (NUE). The objective of this experiment was to optimize N application and SR to regulate wheat growth and increase both GY and NU_E_. The results revealed that net photosynthetic rate (Pn), stomatal conductance (Gs), chlorophyll content, and activities of metabolic enzymes (NR and GS) significantly increased with increasing of N levels while decreasing SR. Plant tillers, GY, DM before anthesis, and N translocation, N agronomic efficiency (NA_E_), N recovery efficiency (NR_E_), and N uptake efficiency (NUP_E_) were highest in a combined treatment of N_235_ and SR_180_. However, N levels beyond 235 kg ha^−1^ significantly decreased NA_E_, NR_E_, and NUP_E_. By increasing SR from 135 to 180 kg ha^−1^ an increase of 12.9 % and 9.1% GY and NUPE, respectively, was observed. Based on this result, we estimate that 1 kg N ha^−1^ might be replaced by an increase of approximately 0.6 kg ha^−1^ SR. Our study suggested that using a combination of N and SR (N_235_ + SR_180_) could attain maximum GY and improve NU_E_ parameters.

## 1. Introduction

Wheat is the main staple crop globally and plays a crucial role in challenging food security, with a total production of 736.1 million tons. Wheat grain yield is not only dependent on genetic potential (variety) and environmental constraints [1] but also depends on management practices [2,3]. Nitrogen is the essential nutrient for wheat growth and production [4,5] which is necessary for maintaining plant growth, biomass, and grain yield [6]. N deficiency in cereal crops reduces fertile tiller numbers [7,8,9], grain number, and kernel weight [10,11]. However, overuse of N results in environmental problems including N leaching, runoff, and volatilization [12], and reduces overall N use efficiency (NU_E_) [13]. In China, a rapid increase in wheat yield was in parallel with the dramatic use of N fertilizer since the 1950s [14]. Importantly, in the last 20 years, N input beyond the threshold level only caused prolonged yield improvement while severe environmental pollution [12,15,16]. Thus, the China government proposed the Double Reduction Plan [15]. A low dose of N with high NU_E_ must be one of the main research goals in plant nutrition [16].

However, enhancing crop profitability and NU_E_ simultaneously is necessary for sustainable agriculture [17] and it is a key challenge to improve viable agriculture in the next decades [18]. N fertilizer is often widely overused to obtain ideal productivity while the adjustment of SR is neglected, which usually synchronously improves productivity and NU_E_. When the N rate was decreased or cut, the output would be lost by reducing tillering and fertile tillers, grain number, and kernel weight [19]. The increase in SR could partially compensate for the decrease in fertile tillers and spike numbers and final productivity [20]. Increasing plant density from 135 to 405 plants m^−2^ [21] or 75 to 300 plants m^−2^ [11] significantly increased grain yield and other parameters. However, there must be an optimum SR to compensate for the negative effects of decreasing N for balanced high yields and improved NU_E_ in wheat. Therefore, it is necessary to investigate the compensatory effect of increasing SR on the decreasing N input on wheat productivity and N use efficiency. It is also necessary to clarify their combination effects on the physiological and agronomical performance of wheat to reveal the underlying rules for balanced high grain yield and improved NU_E_ in winter wheat.

A field experiment with different N and SR levels in two successive years was carried out to determine the optimum combination of N and the seed rate leading to improvement in grain yield and N use efficiency.

## 2. Results

### 2.1. Physiological Traits

#### 2.1.1. Photosynthetic Capacity, Chlorophyll Content, and Leaf Area Index

**Figure 1:**
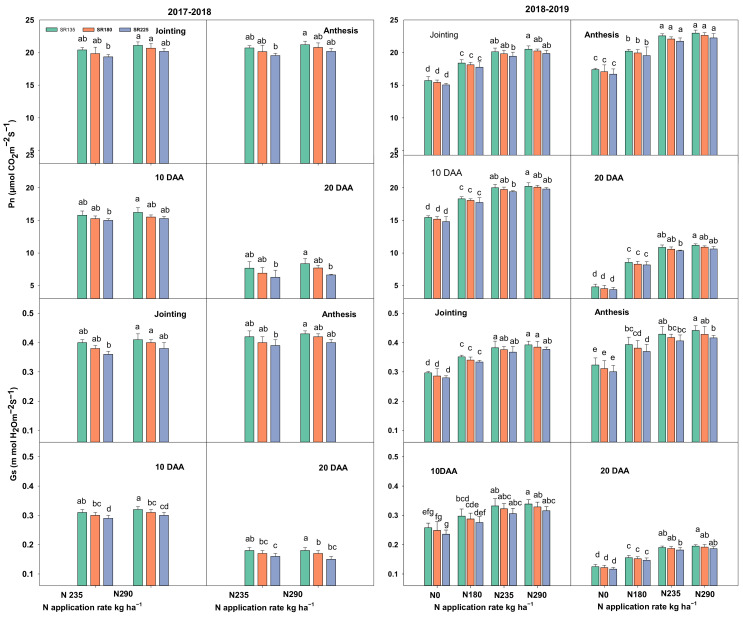
Effects of the combination of N and SR on photosynthesis (Pn) and stomatal conductance (Gs) during four differnet growing stages in 2017–2018 and 2018–2019. Different letters represent significant differences in mean values of three replicate plots at *p* ≤ 0.05 levels according to Duncan’s test.

**Figure 2:**
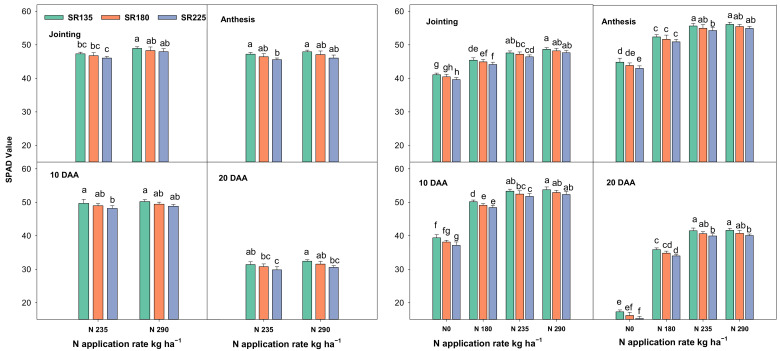
Effects of N and SR on chlorophyll content (SPAD) during four differnet growing stages in 2017–2018 and 2018–2019. Different letters represent significant differences in mean values of three replicate plots at *p* ≤ 0.05 levels according to Duncan’s test.

**Figure 3:**
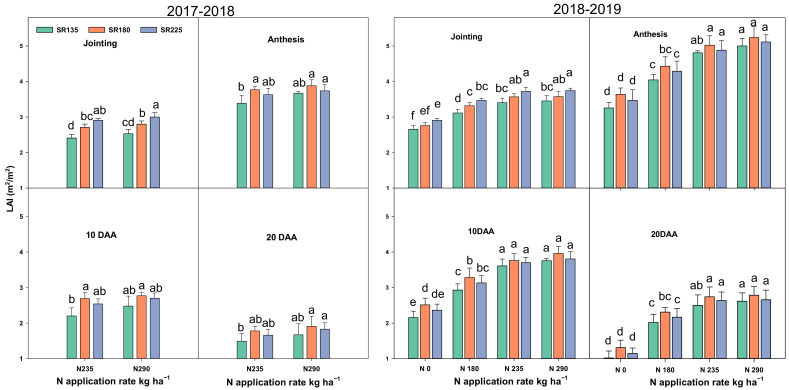
Effects of the combination of N and SR on the values of the leaf area index (LAI) during four differnet growing stages in 2017–2018 and 2018–2019. Different letters represent significant differences in mean values of three replicate plots at *p* ≤ 0.05 levels according to Duncan’s test.

Pn, Gs, SPAD, and LAI were highest at the anthesis stage, followed by the jointing stage, 10 days after anthesis (10 DAA) and 20 DAA, respectively. N application and SR have significant effects on various physiological traits, viz., leaf photosynthetic capacity, chlorophyll content (SPAD value), as well as leaf area index (LAI) at all growth stages (Figure 1, Figure 2 and Figure 3). Photosynthetic capacity (Pn), stomatal conductance (Gs), and SPAD were significantly increased by increasing the N rate or decreasing SR. A significant increment was observed as the N application rate increased from 0 to 235 kg ha^−1^. When N increased from 235 to 290 kg ha^−1^, there was no significant increase for Pn, Gs, and SPAD in both growing seasons. The Pn, Gs, and SPAD values were decreased significantly when SR increased from 135 to 225 kg ha^−1^ in both growing seasons at all sampling stages. Moreover, LAI was significantly increased with an increase in both factors (N + SR). A significant effect was observed when N increased up to N_235_ treatment. There was no significant difference between the N_235_ and N_290_ treatments in LAI in both growing seasons (Figure 3). SR also significantly increased the LAI in both growing seasons from SR_135_ to SR_180_, beyond this level there was no significant effect.

#### 2.1.2. Enzymatic Activities of NR and GS

**Figure 4:**
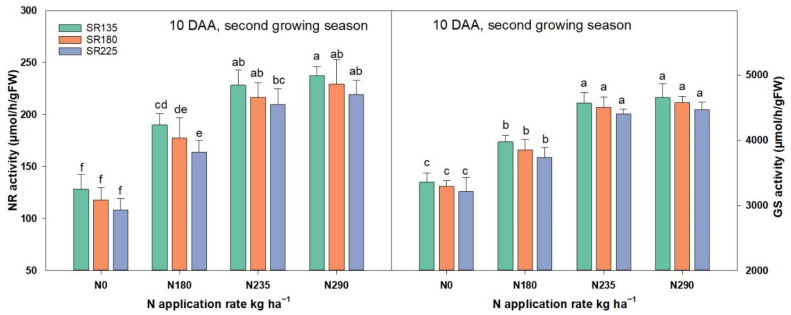
Combination effects of N and SR on nitrate reductase (NR) and glutamine synthesize (GS) activities during four differnet growing stages in 2018–2019. Different letters represent significant differences in mean values of three replicate plots at *p* ≤ 0.05 levels according to Duncan’s test; * *p* ≤ 0.05 and ** *p* ≤ 0.001, respectively.

Nitrate reductase (NR) and glutamine synthesize (GS) play an essential role in N metabolism assimilation and regulation. NR and GS′s enzyme activities were significantly increased by increasing N and decreasing SR (Figure 4). However, the activities of NR and GS differed at the various levels of N application. Furthermore, there was a significant variation in NR and GS activities between N_0_, N_180_, and N_235_, whereas there was no significant difference between the N_235_ and N_290_ treatments. The highest value of the NR and GS activities appeared as the combination of N_290_ and SR_135_ treatments (Figure 4).

### 2.2. Grain Yield and Related Agronomic Characteristics

The grain yield (GY) was significantly influenced by N level and seed rate (N and SR), as well as by their interaction in both years (Table 1). With the increase in the N application rate the GY significantly increased. At SR of 180 kg ha^−1^, GY increased from 6.7 to 8.3 t ha^−1^ when N application increased from N_180_ to N_235_ kg ha^−1^ in 2018–2019. There was no significant difference between N_290_ and N_235_ kg ha^−1^ in both growing seasons. SR significantly affected GY in both growing seasons. Under the same amount of N application (N_235_ kg ha^−1^), GY increased from 5 to 5.7 t ha^−1^ in the first growing season and from 7.5 to 8.3 t ha^−1^ in the second growing season (Table 1). The highest GY 8.3 t ha^−1^ was obtained with the combination of N_235_ + SR_180_ kg ha^−1^ in 2018–2019. N and SR significantly affected the agronomic parameters viz., number of spikes (NS), 1000-grain weight (TGW), number of grains per spike, harvest index (HI), and plant height (PH). All agronomic parameters (NS, TGW, NGS, HI, and PH) were significantly increased with the increasing N application rate. Compared to the treatment with SR_180_ N_0_, NS was significantly increased by 13.2%, 23.8%, and 22.4 % and NGS was increased by 21.8%, 36.8%, and 37.8% in treatments SR_180_N_180_, SR_180_N_235_, and SR_180_N_290_, respectively, in the second growing season. Except for the first growing season in which PH was scarcely increased, TGW and HI were decreased when N application rate increased from N_235_ to N_290_. At the second growing season, none of the agronomic characteristics were significantly influenced beyond N_235_ kg ha^−1^.

SR significantly influenced all of the agronomic parameters. Increasing SR significantly increased NS, HI, and PH during both growing seasons and decreased NGS and TGW. As an example, compared with the N_235_SR_135_ treatment, NS was significantly increased by 10.8% and 11.6%, while NGS was decreased by 4.5% and 6.7% in treatments N_235_SR_180_ and N_235_SR_225_, respectively, in the second growing season (Table 1).

**Figure 5:**
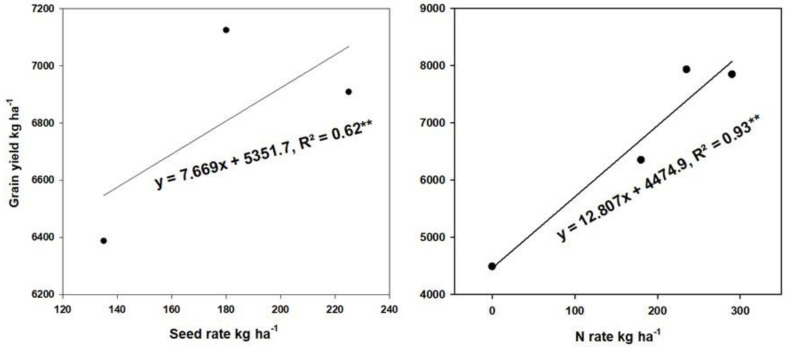
Estimating the regression line to compare N and seed rate for balancing grain yield and improving NU_E_. * *p* ≤ 0.05 and ** *p* ≤ 0.001, respectively. The dots represent the mean value of grain yield under each seed rate (left) or nitrogen rate (right).

Increasing the application of N increased the fodder part and the yield of wheat. Increasing the seed rate can increase the spike number and compensate for reducing the N application rate. Linear regression was used to assess replacing N with SR for balancing GY and NU_E_ parameters. According to the equations obtained from linear regression, the increase in grain yield by 1 ton ha^−1^ needs to increase the seed rate by 7669 kg ha^−1^ or increase the fertilization with N by 12,807 kg ha^−1^. This means that 7669 kg ha^−1^ seed rate is equivalent to 12,807 kg N; therefore, 0.598 kg ha^−1^ seed rate would approximately replace 1 kg of N ha^−1^ (Figure 5). Based on our result, increasing SR from 135 to 180 kg ha^−1^ was observed to increase by 12.9% and 9.1% for GY and NUPE, respectively.

### 2.3. Accumulation, Translation, and Partitioning of DM

DM accumulation (DMA) was significantly affected by the rate of N application and SR (Table 2). Increasing the rate of application of N significantly increased DMA in all growth stages. The highest amount of DMA appeared during the jointing to the anthesis stage. SR significantly increased DMA at all growth stages. The significant effects of SR were up to 180 kg ha^−1^, and beyond SR_180_ kg ha^−1^ was not further influenced (Table 2). Similarly, when N fertilizer increased beyond N_235_ kg ha^−1^, the values of DMA were not significantly increased. At maturity, the highest increase in DM was found with N_235_SR_180_ treatment.

The translocation and contribution of DM were significantly affected by the application rate of N and SR (Table 2). With increasing N rate, pre-anthesis translocation (PTA), post-anthesis accumulation (PAA), and contribution of post-anthesis to grain (CPA) were significantly increased, while the contribution of pre-anthesis translocation to grain (CPT) was significantly decreased. The maximum value of PTA appeared in the N_235_ treatment, which was significantly higher than the N_290_ treatment. Furthermore, the value of PTA increased with increasing SR, while PAA and CPA significantly increased up to SR_180_ kg ha^−1^. The CPT values first decreased and then increased as the SR increased.

The partition of DM into different parts of the plant differed between the N and SR treatments (Table 3). At the anthesis stage, the DM of culm + sheath was higher than the DM of rachis + glumes and the DM of rachis + glumes was higher than the DM of the leaves. However, at the harvesting stage, the grain DM was higher than the DM of the culm + sheath, the DM of culms + sheaths was higher than the DM of rachis + glumes, and the DM of the rachis + glumes was higher than the DM of leaves. The proportion of distribution of DM of grains, rachis + glumes, culms + sheathes, and leaves ranged from 37.1% to 45.8%, 12.7% to 14.1%, 33% to 41.4%, and 7.8% to 8.5%, respectively, at harvesting.

### 2.4. Accumulation, Translocation, and Partitioning of N

Accumulation of N (NA) at all parts of the plant was significantly increased by increasing N application and SR up to certain levels (N_235_, SR_180_) at both anthesis and maturity stages. At the maturity stage, the total content of N compared to control (N_0_) treatment increased by 76.9%, 134%, and 139% in the treatments N_180_, N_235_, and N_290_. The N content compared to SR_135_ was increased by 8.8% and 5% in the SR_180_ and SR_225_ treatments (Table 4). As well, N translocation before anthesis to grain (NTA), N accumulation after anthesis (NAA), and the contribution rate of NA after anthesis to grain (CAG) were significantly increased with the increase in N application. NTA increased 32.18% and 2.9% when N increased from N_180_ to N_235_ from N_235_ to N_290_, respectively (average of three SR treatments). Furthermore, the contribution rate of pre-N translocation to grain (CTG) had the same trend as CPT. Furthermore, by increase in SR up to 180 kg ha^−1^, NTA, NAA, and CAG were significantly increased, while CTG was at first significantly decreased then increased (Table 4).

Plant N partitioning was also influenced by N and SR. Compared with the control treatment (N_0_), the N_180_, N_235_, and N_290_ treatments increased the N content by 53.8%, 153%, and 144% in the rachis + glumes, by 84.2%, 149.2%, and 157.6% in the culms + sheathes, and by 65.8%, 107.7%, and 109.4% in the part of leaves, respectively, at anthesis stage. However, this increase in N at the harvesting stage was by 78.7%, 140.3%, and 145.6% for grains, by 52.9%, 98.5%, and 103% for rachis + glumes, by 61.5%, 88.1%, and 99% for culms + sheathes, and by 113.7%, 182%, and 186% for leaves in treatments N_180_, N_235_, and N_290_ compared to N0 treatment, respectively. Additionally, compared to the SR_135_ treatment, the SR_180_ and SR_225_ treatments increased N content by 8.6% and 1% at the rachis + glumes, by 1.7% and 2% at the culms + sheathes, and by 16% and 3.7% at the parts of leaves, respectively, at anthesis stage. Compared to SR_135_ treatment, the N content of the SR_180_ and SR_225_ treatments increased by 9.8% and 6.2% in grains, by 5.5% and 1.8% in the rachis + glumes, by 5.1% and −2% in the culms + sheathes, and by 5.5% and 2.7% in the leaves, respectively, at the maturity stage (Table 5). The range of the N distribution ratio of different parts, i.e., rachis + glumes, culms + sheathes, and leaves were from 17% to 21.9%, from 39.1% to 47.1%, and from 33.5% to 41.6% at anthesis, respectively. The maturity stage range of N distribution range was 75.4% to 79.2%, from 5.8% to 7.4%, 8.8% to 11.9%, and from 5% to 6.4% for grains, rachis + glumes, culms + sheathes, and leaves, respectively (Table 5).

### 2.5. N Use Efficiency (NU_E_) Parameters

N rates, SR, and their interaction had a significant effect on N agronomy efficiency (NA_E_), N uptake efficiency (NUP_E_), and N partial factor productivity (NPFP). Increasing N level up to N_235_ kg ha^−1^, NA_E_, NUP_E_, N recovery efficiency (NR_E_), and N harvest index (NHI) were significantly increased. However, increasing N levels beyond N_235_ kg ha^−1^, NA_E_, NR_E_, and NUP_E_ were significantly decreased. The NPFP values were decreased at all N levels. Furthermore, the NA_E_, NR_E_, NUP_E_, NPFP, and NHI values decreased by 12.8%, 10.4%, 17.3%, 13.6%, and 0.4%, respectively, at N_290_ treatment compared to N_235_ treatment. Similarly, SR had a considerable effect on the NU_E_ parameters, but the effect of SR was less compared to the N treatment. Maximum values for the parameters NA_E_, NR_E_, and NUP_E_ were observed from the combination of treatment with N_235_ and SR_180_ (Table 6).

### 2.6. Correlation Analysis

#### 2.6.1. Correlation of GY with Agronomic and Photosynthesis Traits

The key relationships between the GY-related parameter variables are shown in Table 7. There was a significant positive correlation between GY and NS, NGS, TGW, PH, HI, Pn, Gs, SPAD value, and LAI. However, there was no significant relationship between GY and PH (Table 7).

#### 2.6.2. Relationship between GY and Enzyme Activities and NUE Parameters

**Figure 6:**
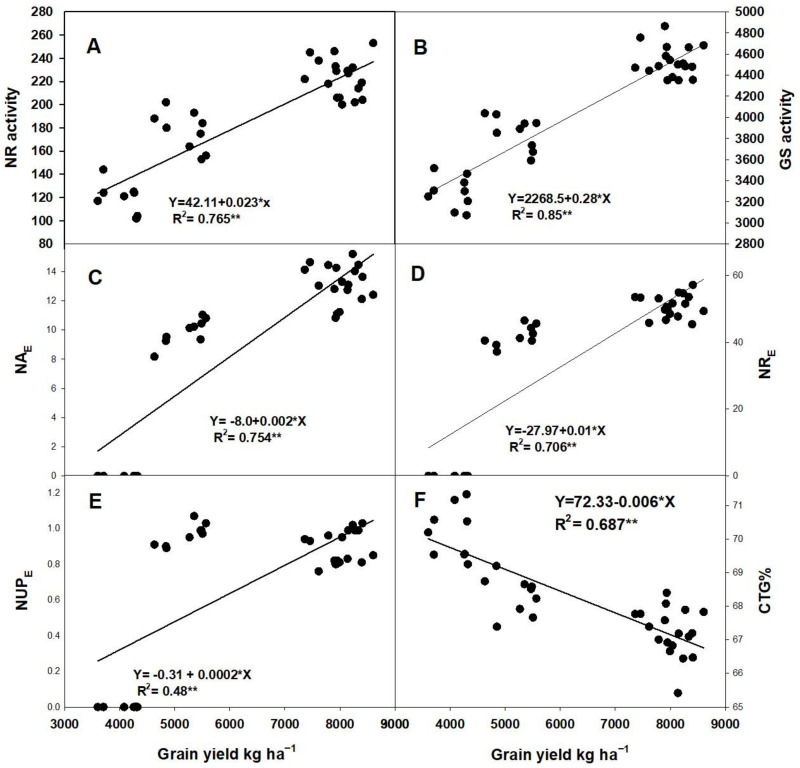
Regression analyses among GY and nitrate reductase (NR)/(**A**) glutamine synthesis (GS)/(P**B**), N agronomy efficiency (NAE)/(**C**), N recovery efficiency (NR_E_)/(**D**), N uptake efficiency (NUP_E_)/(**E**), and contribution of N translocation to the grain after the anthesis stage (CTG)/(**F**), respectively. * *p* ≤ 0.05 and ** *p* ≤ 0.001, respectively.

Grain yield had a significant and positive relationship with nitrogen reductase (NR) and glutamine synthesis (GS) enzymes, N agronomic efficiency (NA_E_), N recovery efficiency (NR_E_), and N uptake efficiency (NUP_E_). However, GY had a significantly negative relationship with N translocation’s contribution before anthesis to grain (CTG) (Figure 6).

Increasing N application from 235 to 290 kg ha^−1^, GY did not increase significantly (0.4%) while NA_E_, NR_E_, and NUP_E_ were decreased 12.8%, 10.4%, and 17.3%, respectively. Maximum GY and highest values of NU_E_ parameters, particularly NA_E_, NR_E_, and NUP_E_, were observed from the combination of treatment with N_235_ and SR_180_ (Table 1 and Table 6).

## 3. Discussion

DM and N translocation are well-known to greatly contribute to the final GY [22,23]. However, there is a lack of research on the combined effects of N and SR on the DM and N translocation, NU_E_ parameters, growth physiological parameters, and their relationship with GY in winter wheat. The effects of the excessive rate of N and N′s compensation by increasing SR on final GY and NU_E_ parameters were unknown.

### 3.1. Physiological Characteristics

The plant leaf′s photosynthesis capacity plays a crucial role in plant growth and grain yield [24], and approximately 70% of productivity is derived from post-anthesis photosynthesis. In the present study, increasing N application and decreasing SR, resulted in a significantly increased Pn, Gs, and SPAD values in both growing seasons. Furthermore, LAI was significantly increased by increasing N application and SR (Figure 1, Figure 2 and Figure 3). However, it has shown that the maximum values for Pn, Gs, and SPAD parameters were exhibited from the combination of N_290_ + SR_135_ treatment but there were no significant differences between the mentioned and N_235_ + SR_180_ treatment. The main reason for decreasing the Pn, Gs, and SPAD values by increasing SR might be due to more competition and the over-crowded shading effect as reported by [25]. In addition, N reductase (NR) and glutamine synthesize (GS) increased significantly with the N application rate and the declaration of SR. Notably, there was no significant difference for the value of both mentioned enzyme activities when N fertilizer amount increased from N_235_ to N_290_. These findings suggest N and SR′s optimization benefits for maintaining strong photosynthesis capacity and N assimilation ability in wheat plants.

### 3.2. Grain Yield (GY) and N Use Efficiency (NU_E_)

Simultaneous improvement in GY and NU_E_ of wheat is an important objective in modern agriculture management. Here, we investigated the suitable combinations of N and SR to obtain higher GY and NUE. The maximum value was observed with the treatment of N_235_ + SR_180_ kg ha^−1^ in both growing seasons. The previous finding can explain that too high plant density had no significant effect on wheat grain yield [11,26]. According to our results, yield loss caused by reducing the N rate can be compensated by increasing SR (Figure 2). It was estimated by a linear regression that every decrement of 1 kg N ha^−1^ can be replaced by adding 0.6 kg ha^−1^ SR. The GY obtained by adding SR is clearly attributed to the increasing spike number (SN) (Table 1). Moreover, the yield in 2017–2018 (Y1) was much lower than that in 2018–2019 (Y2), which may be due to the adverse weather conditions. There was excessive rainfall and less sunshine during grain filling in the first growing season (Figure 1), leading to lower yield components and GY [1].

NU_E_ results from the incorporation of N-uptake efficiency (NUP_E_) and N-utilization efficiency (NUT_E_) [27]. In detail, NUP_E_ the plant’s capacity to extract N from the soil, and depending on the root structure and the relation of N transporters [28]. In this study, increasing N application from N_235_ to N_290_ resulted in a decrease in NAE, NR_E_, NUP_E_, and NPFP by 12.8%, 10.4%, 17.3%, and 13.6%, respectively. Similarly, a result was obtained by [29] that treatment with N_240_ and N_300_ compared to treatment with N180, NPFP were decreased by 24.5% and 37.4% and NA_E_ were decreased by 23.5% and 31.9%, respectively. The decrease in NU_E_ after the optimal rate might be due to more losses by increasing N application according to the previous finding of [11]. Here, our results further confirmed that NU_E_ components were significantly increased up to a certain amount of SR (180 kg ha^−1^), which is in good agreement with the previous study [30]. This increase in the NU_E_ response to the high SR could be due to an increase in the density of the roots in the soil, which enhanced N from deeper parts of the soil. Therefore, it is not surprising that maximum values of NA_E_, NR_E_, and NUP_E_ were also observed from the optimal combined treatment (N_235_ + SR_180_), which was also the case for grain yield (Table 2). Our study thereby provides a practical management method approaching higher GY and NUP_E_ by the optimization of N and SR.

### 3.3. Accumulation and Translocation of DM and N

Total dry matter accumulation and partitioning into separate parts of the plant were significantly affected by combined N and SR′s combined treatments. The maximum value of total dry matter and individual parts especially grains, rachis + glumes, and leaves, was also obtained from the combination of N_235_ + SR_180_ treatment. Recent studies reported that the contribution of pre-anthesis translocation to grain was significantly decreased when the N application rate increased [31,32]. Similarly, the result of the current experiment showed that the contribution of pre-anthesis DM translocation was significantly decreased with increasing N rate, while pre-anthesis DM translocation, post-anthesis DM accumulation, and contribution of post-anthesis DM accumulation to grain were significantly increased. The main reason for the decreasing contribution of pre-anthesis DM translocation to grain might be that early senescence occurred due to N deficiency, which would speed up the pre-translocation from leaf and stem to spike. Furthermore, increasing seed rate significantly increased pre-anthesis dry matter translocation, post-anthesis DM accumulation, and contribution of post-anthesis DM accumulation to the grain. Parallel to our finding, it was reported that post-heading DM and N accumulation was significantly increased with increasing SR [33]. It should be noted that an excessive amount of N application (N_290_ kg ha^−1^) as well as SR (SR_225_ kg ha^−1^) did not increase the amount of DM translocation.

Total N accumulation, partitioning, and translocation showed the same trend with the above part of DM. Both N and SR up to optimal levels (N_235_, SR_180_) significantly increased the total N content of individual parts. The same finding reported that no further increase was observed in the uptake of N at N fertilizer and the density of the plant beyond 240 kg N ha^−1^ and 405 plants m^−2^ [21,34]. N translocation, postanthesis N accumulation, and postanthesis N accumulation contribution of post-anthesis N accumulation to the grain were higher in the high N treatments compared to control and low N treatment. A similar result was found that N translocation and post-anthesis N accumulation were enhanced with increasing N application rate [23]. In the current experiment, N translocation, N accumulation after anthesis, and contribution of post-anthesis to grain response to seed rate were significantly increased from SR _135_ to SR _180_ kg ha^−1^.

### 3.4. Relationship of GY with Related Parameters

Our results showed that GY has a significant and positive correlation with Pn, Gs, SPAD value, LAI, and other GY components (Table 7). This is similar to the results from the study by Jiang et al. [24]. Furthermore, the regression analyses also revealed that GY had a positive correlation with NR, GS, NA_E_, NR_E_, and NUP_E_ while showing a negative correlation with CTG (Figure 6). It was also observed that NR and GS activities were highly positively correlated with photosynthesis capacity, which is consistent with previous studies [35,36]. Here, we found that GY had a significant positive correlation with NU_E_ parameters such as NA_E_, NRE, and NUP_E_. This was not in agreement with the previous finding that GY showed a negative correlation with NU_E_ [37,38]. The reason might be due to a certain amount of N + SR (N_235_ + SR_180_), in which both NU_E_ and GY were significantly higher up to the previous partnership. In conclusion, we found that N and SR′s improper rate cannot increase GY, but significantly decreased NU_E_. In this regard, to achieve the maximum GY and NU_E_, it would be better to use the optimal amount of both N and SR, which is the result of the current experiment, and the suitable combined treatment was N_235_ + SR_180_. Furthermore, by using a suitable combination of N and SR (SR_180_ N_235_), replacing N to SR especially for balancing GY and NU_E_ would be the best method for sustainable agriculture. According to our findings, we infer that, based on low SR (SR_135_), 1 kg ha^−1^ N could be saved by increasing approximately 0.6 kg ha^−1^ SR.

## 4. Materials and Methods

### 4.1. Plant Material and Experimental Site

The field experiment was conducted during two successive growing seasons (2017–18 and 2018–19) at the XuYi Rice and Wheat demonstration center (118°43′ N and latitude 32°59′ E) in Jiangsu province, China. The winter wheat cultivar Ningmai 13 was used in both growing seasons. The soil type was clay loam and the pH was 6.8. It contained 31.07 g kg^−1^ of organic matter, 2449 g kg^−1^ of available N, 27.3 mg kg^−1^ of available phosphate, and 240 mg kg^−1^ of available potassium. Seeds were sown on 31 October 2017, and 1 November 2018, and the crop was harvested on 3 June 2018, and 6 June 2019, respectively.

### 4.2. Experiment Design

The experiment was carried out according to the split-plot design with three replicates. The main plot consisted of three seed rates (SR_135_, SR_180_, and SR_225_ kg ha^−1^) and the subplot comprised two levels of doses of N in the first year (N_235_ and N_290_ kg ha^−1^) and three N levels in second year of the experiment (N_180_, N_235_, and N_290_ kg ha^−1^). At the first growing season, there was no significant effect between N_235_ and N_290_ for GY, because of that we added N_180_ kg ha^−1^ treatment at the second growing season to determine the N effect as well to determine the optimum rate of N for GY. An N-control plot (N_0_) was also used at the second growing season per replication for the calculation of NU_E_ parameters. N fertilizer was applied as urea (46%), phosphorus (P) and potassium (K) fertilizer as calcium superphosphate (15%), and potassium chloride (60%) at the rates of 120 (P_2_O_5_) and 120 (K_2_O) kg ha^−1^. All of the phosphorus and potassium fertilizer and 70% of the total amount of N fertilizer were spread by hand before plowing at the time of sowing. The remaining N fertilizer (30%) was applied at the first node (31) according to BBCH. Each plot size was 4 × 3 m and consisted of 12 rows with a row-to-row distance of 25 cm.

In this experiment, the rice–wheat rotation system was undertaken for the long term. Rice cultivation techniques such as puddling, transplanting, and flooding, and the whole amount of straw returned to the field almost in the last decade. Only 5 cm of rice straw remains above the ground. Moldboard plough was followed by rotary plough as primary and secondly tillage. The depth of moldboard tillage was 20 cm and that of the rotary plough was 10 cm. Wheat seeds were sown with a seed drill precise machine with surface stubble plowing and roll compaction. For high-yield production, insects, diseases, and weeds were controlled two times by spraying insecticide (Biscaya), fungicide (Capalo), and herbicide (sulfosulfuron) during both growing seasons. 

### 4.3. Grain Yield and Yield Components

Uniform plants at the flowering stage were tagged with labels and were sampled at a later stage. At the maturity stage, ears/spikes f rom the area of 0.5 m^2^ (without taking a sample) of each plot were collected to determine GY and yield components.

### 4.4. Photosynthesis, SPAD, LAI, N, and Enzyme Activities in Leaves

Photosynthesis (Pn), stomatal conductance (Gs), SPAD, and LAI were measured at the jointing stage, anthesis, 10 days after anthesis (DAA), and 20 DAA. Pn and GS were measured by a portable gas exchange analyzer (LI-6400XT;LI-COR-Inc., Lincoln NE, USA) at 9:00–11:00 a.m. on a sunny day. The concentration of CO_2_ in the leaf chamber, light intensity, and relative humidity were set as 380 μmol mol^−1^, 1000–1100 µmol m^−2^s^−1^, and 500 mL min ^−1^, respectively. The SPAD value was determined by Minolta 502 chlorophyll meter (Minolta, Japan). LAI was measured with using a leaf area meter (LI-3100, LI-COR, Lincoln, NE, USA). N concentration was determined by the micro Kjeldahl method [39]. Nitrate reductase (NR) and glutamine synthesis (GS) were determined according to the method previously described by [36] and [40].

### 4.5. Dry Matter (DM) and N Translocation

DM and N accumulation (DMA and NA), translocation, and their contribution were estimated according to [31] and [41] by using the following equations (Table 8).

### 4.6. Use Efficiency (NU_E_) Parameters

NU_E_ parameters were determined using the following equations described by [17], and [42] (Table 9).

### 4.7. Weather Condition

**Figure 7:**
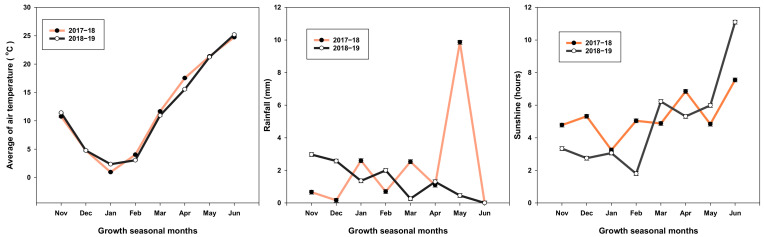
Metrological data: (monthly average temperature, rainfall (mm), and sunshine per hour) in two successive growing season.

Monthly average temperature, rainfall, and sunshine at the experimental site over two successive years (2017–2018 and 2018–2019) are presented in Figure 7. There was considerable variation between the two growing seasons. At the active tiller stage (from late January to the end of the first week of February), the minimum temperature in 2017–2018 was lower (−4.5 °C) compared to that of the second growing season (−0.5 °C). At the anthesis stage, the average rainfall in the first growing season was 538 mm, which was 64.02% higher than that in the second growing season (328 mm).

### 4.8. Statistical Analysis

Two-way ANOVA (SPSS version 17.1) was used for analyzing the variance among different treatments. The means were tested with the least significant difference at the 0.05 probability level (*p* ≤ 0.05 by Duncan’s). Pearson’s correlation between grain yield and related parameters were calculated through the SPSS version17.1. All graphs and linear regression analyses was done by sigmaplot 14.0 software (Chicago, IL, USA).

## 5. Conclusions

In summary, the GY, DMA, NAC, NU_E_ parameters and physiological parameters increased significantly with the combination of N_235_ and SR_180_. However, the excessive rate of N application cannot increase GY and other parts of plant DM but it decreased NA_E_, NR_E_, and NUP_E_. Our result confirmed that maximum GY and higher NU_E_ components could be achieved via avoiding excessive use of N, and optimizing the compensation effect of increasing SR for reducing N application.

## Figures and Tables

**Table 1:** Effects of N and SR on GY and agronomic characteristics in 2017–2018 and 2018–2019.

	N kg ha^−1^	SR kg ha^−1^	TY t ha^−1^	NS × 10^4^ ha^−1^	TGW(g)	NGS	HI	PH (cm)
**2017–2018**	N_235_	SR_135_	5.05 ^b^	357 ^c^	39.7 ^a^	35.63 ^a^	0.41 ^c^	73.4 ^c^
		SR_180_	5.78 ^a^	414 ^b^	39.5 ^ab^	35.38 ^a^	0.44 ^ab^	74.33 ^bc^
		SR_225_	5.7 ^a^	425 ^ab^	39.2 ^bc^	34.27 ^b^	0.45 ^a^	75.74 ^ab^
	N_290_	SR_135_	5.03 ^b^	361 ^c^	39.1 ^bc^	35.63 ^a^	0.41 ^c^	76.43 ^ab^
		SR_180_	5.77 ^a^	420 ^ab^	39 ^bc^	35.3 ^a^	0.43 ^bc^	77.23 ^a^
		SR_225_	5.77 ^a^	434 ^a^	38.9 ^c^	34.18 ^b^	0.42 ^bc^	77.57 ^a^
	F-Value	N	0.113	2.475	13.65 **	0.098	7.478 *	19.88 **
		SR	107.1 **	105.9 **	3.38 *	21.65 **	11.46 **	2.999
		N*S	0.331	0.123	0.63	0.025	1.637	0.426
	N_0_	SR_135_	4 ^g^	391 ^g^	35.7 ^bc^	28.8 ^d^	0.35 ^e^	62.93 ^d^
		SR_180_	4.8 ^f^	479 ^ef^	35.6 ^bc^	28.1 ^d^	0.4 ^cde^	63.62 ^d^
		SR_225_	4.8 ^f^	503 ^de^	35.5 ^c^	27.2 ^d^	0.4 ^cd^	64.07 ^d^
2018–2019	N_180_	SR_135_	5.6 ^e^	448 ^f^	36.4 ^ab^	34.5 ^c^	0.38 ^de^	72.87 ^c^
		SR_180_	6.7 ^d^	543 ^bc^	36.3 ^abc^	34.1 ^c^	0.41 ^cd^	74.02 ^bc^
		SR_225_	6.6 ^d^	555 ^b^	36.3 ^abc^	33.1 ^c^	0.42 ^bc^	74.47 ^b^
	N_235_	SR_135_	7.5 ^c^	518 ^cd^	36.7 ^a^	39.6 ^ab^	0.45 ^ab^	73.97 ^bc^
		SR_180_	8.3 ^a^	593 ^a^	36.7 ^a^	38.3 ^ab^	0.46 ^a^	75.15 ^ab^
		SR_225_	8.2 ^a^	597 ^a^	36.5 ^ab^	37.5 ^b^	0.46 ^a^	75.75 ^a^
	N_290_	SR_135_	7.8 ^bc^	529 ^bc^	36.6 ^a^	40.4 ^a^	0.45 ^ab^	73.95 ^bc^
		SR_180_	8.2 ^a^	587 ^a^	36.4 ^ab^	38.6 ^ab^	0.44 ^ab^	75.22 ^ab^
		SR_225_	8.1 ^ab^	591 ^a^	36.4 ^ab^	37.7 ^ab^	0.45 ^ab^	75.97 ^a^
	F-Value	N	690 **	74.4 **	9.66 **	242.5 **	43.55 **	598.3 **
		SR	60.1 **	83.94 **	0.66	12.19 **	4.48 *	17.8 **
		N*SR	2.64 *	1.36	0.027	0.394	1.77	0.25

Note: N, SR, TY, NS, TGW, NGS, HI, and PH indicate nitrogen application, seed rate, theoretical yield number of spikes, thousand-grain weight, the number of grains per spike, harvest index, and plant height, respectively. Different letters in the same column represent significant differences in mean values of three replicate plots according to Duncan’s test; * *p* ≤ 0.05 and ** *p* ≤ 0.001, respectively.

**Table 2:** Combination effects of N and SR on DM accumulation, translocation in 2018–2019.

Total DM Accumulation kg ha^−1^					DM Translocation				
**N kg ha^−1^**	**SR kg ha^−1^**	**So-JT**	**JT-An**	**An-M**	**So-M**	**PTA kg ha^−1^**	**CPT%**	**PAA kg ha^−1^**	**CPA%**
N_0_	SR_135_	1959 ^h^	6139 ^b^	2680 ^c^	10778 ^g^	1214.1 ^f^	30.3 ^a^	4256 ^d^	69.7 ^e^
	SR_180_	2314 ^g^	6514 ^b^	3250 ^bc^	12079 ^f^	1322.8 ^f^	27.6 ^b^	5072 ^d^	72.4 ^d^
	SR_225_	2401 ^f^	6682 ^b^	3025 bc	12108 ^f^	1439 ^e^	29.7 ^a^	5018 ^d^	70.3 ^e^
N_180_	SR_135_	2395 ^f^	8585 ^a^	3708 ^b^	14689 ^e^	1529.4 ^de^	27.2 ^bc^	6053 ^c^	72.8 ^cd^
	SR_180_	2856 ^b^	8904 ^a^	4780 ^a^	16539 ^cd^	1633.8 ^cd^	24.3 ^d^	7276 ^b^	75.7 ^b^
	SR_225_	2488 ^e^	8587 ^a^	4646 ^a^	15721 ^d^	1701.4 ^c^	25.6 ^cd^	7164 ^b^	74.4 ^bc^
N_235_	SR_135_	2668 ^d^	9335 ^a^	4731 ^a^	16735 ^c^	1918.7 ^ab^	25.5 ^cd^	7804 ^ab^	74.5 ^bc^
	SR_180_	3019 ^a^	10027 ^a^	5130 ^a^	18177 ^ab^	1963.7 ^a^	23.6 ^de^	8727 ^a^	76.4 ^ab^
	SR_225_	3033 ^a^	9804 ^a^	5064 ^a^	17902 ^ab^	2028.5 ^a^	24.9 ^d^	8490 ^a^	75.1 ^b^
N_290_	SR_135_	2719 ^c^	9477 ^a^	5048 ^a^	17245 ^bc^	1905.8 ^ab^	24.4 ^d^	8141 ^ab^	75.6 ^b^
	SR_180_	2998 ^a^	10066 ^a^	5418 ^a^	18483 ^a^	1823.2 ^b^	22.1 ^e^	8776 ^a^	77.9 ^a^
	SR_225_	3010 ^a^	9649 ^a^	5459 ^a^	18119 ^ab^	1906.7 ^ab^	24 ^de^	8518 ^a^	76.4 ^ab^
F-Value	N	1817 **	28.1 **	32.1 **	258 **	167.9 **	50.4 **	92.454 **	50.4 **
	SR	84 **	0.9	4.3 *	23.8 **	11.1 **	16 **	9.546 **	16 **
	N*SR	67.1 **	0.1	0.3	0.4	1.9	0.2	0.28	0.2

Note: S_O_-JT, JT-An, An-M, S_O_–M, PTA, CPT, PAA, and CPA represent sowing to jointing, jointing to anthesis, anthesis to maturity, sowing to maturity, pre-anthesis DM translocation amount, contribution of pre-anthesis translocation to grain, post-anthesis accumulation amount, and contribution of post-anthesis DM accumulation to grain, respectively. Different letters in the same column represent significant differences in mean values of three replicate plots according to Duncan’s test; * *p* ≤ 0.05 and ** *p* ≤ 0.001, respectively.

**Table 3:** Effects of N and SR on DM accumulation and partitioning at the anthesis and maturity stages in 2018–2019.

N kg ha^−1^	SR kg ha^−1^	Grain		Rachis + Glumes		Culms + Sheaths		Leaves	
		Anthesis	Maturity	Anthesis	Maturity	Anthesis	Maturity	Anthesis	Maturity
N_0_	SR_135_		4005 ^g^	1576 ^c^	1465 ^f^	5442 ^g^	4462 ^c^	1080 ^f^	846 ^f^
	SR_180_		4788 ^f^	1821 ^c^	1606 ^e^	5631 ^f^	4741 ^bc^	1376 ^e^	944 ^e^
	SR_225_		4851 ^f^	1994 ^c^	1606 ^e^	5701 ^f^	4682 ^bc^	1389 ^e^	968 ^e^
N_180_	SR_135_		5623 ^e^	2344 ^bc^	1959 ^d^	7217 ^d^	5932 ^a^	1420 ^e^	1175 ^d^
	SR_180_		6712 ^d^	2496 ^bc^	2198 ^bc^	7426 ^c^	6299 ^a^	1837 ^c^	1330 ^c^
	SR_225_		6647 ^d^	2517 ^bc^	2218 ^bc^	6716 ^e^	5525 ^ab^	1841 ^c^	1330 ^c^
N_235_	SR_135_		7535 ^c^	3072 ^ab^	2187 ^c^	7156 ^d^	5642 ^a^	1775 ^d^	1370 ^b^
	SR_180_		8324 ^a^	3597 ^a^	2366 ^a^	7375 ^c^	6017 ^a^	2075 ^a^	1468 ^a^
	SR_225_		8152 ^ab^	3426 ^ab^	2366 ^a^	7341 ^c^	5919 ^a^	2070 ^a^	1464 ^a^
N_290_	SR_135_		7814 ^bc^	3092 ^ab^	2232 ^b^	7199 ^d^	5807 ^a^	1906 ^b^	1391 ^b^
	SR_180_		8244 ^a^	3358 ^ab^	2355 ^a^	7641 ^a^	6425 ^a^	2066 ^a^	1458 ^a^
	SR_225_		8087 ^ab^	3058 ^ab^	2337 ^a^	7545 ^b^	6241 ^a^	2057 ^a^	1453 ^a^
F-Value	N		690 **	14.8 **	2002 **	2858.3 **	18.4 **	955.9 **	1001 **
	SR		60.1 **	0.92	207 **	96.3 **	2.3	324.9 **	85.5 **
	N*SR		2.64 *	0.17	6.29 **	61.7 **	0.58	11.1 **	2.92 *

Note: N and SR represent nitrogen application and seed rate, respectively. Different letters in the same column represent significant differences of mean values of three replicate plots at *p* ≤ 0.05 levels according to Duncan′s test; * *p* ≤ 0.05 and ** *p* ≤ 0.001, respectively.

**Table 4:** Combination effects of N and SR on total N accumulation and translocation in 2018–2019.

		Total N Accumulation kg ha^−1^				N Translocation			
N kg ha^−1^	SR kg ha^−1^	So-JT	JT-An	An -M	So-M	NTA kg ha^−1^	CTG%	NAA kg ha^−1^	CAG%
N_0_	SR_135_	25.6 ^h^	53.4 ^i^	12.4 ^g^	91.4 k	48.3 ^j^	70.1 ^ab^	27.4 j	29.9 ^de^
	SR_180_	28.5 ^g^	60.4 ^h^	14.5 ^f^	103.4 ^i^	55.5 ^i^	69.4 ^bc^	31.5 ^h^	30.6 ^cd^
	SR_225_	28.8 ^g^	60.4 ^h^	11 ^g^	100.2 ^j^	55.3 ^i^	71 ^a^	29.2 ^i^	29 ^e^
N_180_	SR_135_	41.3 ^f^	97.2 ^g^	23.2 ^e^	161.6 ^h^	84.3 ^h^	68.4 ^cd^	48.9 ^g^	31.6 ^bc^
	SR_180_	45.6 ^e^	108.5 ^e^	30.2 ^cd^	184.3 ^f^	97.8 ^f^	68.2 ^d^	56.5 ^e^	31.8 ^b^
	SR_225_	46.1 ^e^	100.6 ^f^	29.1 ^d^	175.8 ^g^	94.7 ^g^	68.3 ^cd^	54.2 ^f^	31.7 ^bc^
N_235_	SR_135_	57.4 ^d^	135.1 ^d^	29.7 ^cd^	222.2 ^e^	118.1 ^e^	67.5 ^de^	70 ^d^	32.5 ^ab^
	SR1_80_	61.7 ^ab^	145.3 ^a^	31.5 ^c^	238.6 ^ab^	125.9 ^ab^	66.7 ^e^	76.9 ^a^	33.3 ^a^
	SR_225_	61.6 ^ab^	136.3 ^d^	31.5 ^c^	229.4 ^d^	122.1 ^d^	67.3 ^de^	72.7 ^bc^	32.7 ^ab^
N_290_	SR_135_	59 ^c^	140.1 ^b^	31.3 ^c^	230.4 ^d^	123.2 ^cd^	67.8 ^de^	72.1 ^c^	32.2 ^ab^
	SR_180_	61.9 ^a^	145.8 ^a^	34.2 ^b^	241.8 ^a^	126.9 ^a^	66.6 ^e^	77.7 ^a^	33.4 ^a^
	SR_225_	61 ^b^	138 ^c^	36.1 ^a^	235 ^c^	124.6 ^bc^	67.4 ^de^	74.2 ^b^	32.6 ^ab^
F-Value	N	1159 **	2229 **	628.1 **	6418 **	9584 **	44.2 **	436.6 **	44.2 **
	SR	270.4 **	377.9 **	31.8 **	1283 **	195.7 **	5.7 *	115.6 **	5.7 *
	N*SR	7.1 **	31.7 **	8.72 **	44.3 **	15.81 **	1	3.03 *	1

Note: SO-JT, JT-An, An-M, SO–M, NTA, CTG, NAA, and CAG represent jointing, jointing to anthesis, anthesis to maturity, sowing to maturity, pre-anthesis N translocation, contribution rate of N translocation to grain, nitrogen accumulation amount, and contribution rate of N accumulation to grain, respectively. Different letters in the same column represent significant differences in mean values of three replicate plots according to Duncan’s test; * *p* ≤ 0.05 and ** *p* ≤ 0.001, respectively.

**Table 5:** Combination effects of N and SR on N partitioning during anthesis and maturity stages in 2018–2019.

N kg ha^−1^	SR kg ha^−1^	N at Grain		N at Rachis + Glumes		NA at Culms + Sheaths		N at Leaves	
		Anthesis	Maturity	Anthesis	Maturity	Anthesis	Maturity	Anthesis	Maturity
N_0_	SR_135_		68.9 ^f^	15 ^g^	6.8 ^c^	34.9 ^g^	10.9 ^e^	29.2 ^i^	4.9 ^d^
	SR_180_		80 ^e^	17.1 ^f^	7.1 ^c^	35.1 ^g^	11.2 ^e^	36.8 ^g^	5.2 ^d^
	SR_225_		77.8 ^ef^	18.2 ^f^	6.6 ^c^	34.9 ^g^	10.6 ^e^	36.1 ^h^	5.1 ^d^
N_180_	SR_135_		123.2 ^d^	25.7 ^e^	10 ^b^	65.2 ^e^	18.1 ^cd^	47.6 ^f^	10.3 ^c^
	SR_180_		143.4 ^c^	26.2 ^e^	10.8 ^b^	67.1 ^d^	18.8 ^bc^	60.8 ^e^	11.3 ^b^
	SR_225_		138.5 ^c^	25.1 ^e^	10.3 ^b^	60.6 ^f^	15.9 ^d^	60.9 ^e^	11 ^bc^
N_235_	SR_135_		174.9 ^b^	40.3 ^c^	13.2 ^a^	87.7 ^b^	20.1 ^abc^	64.5 ^d^	14 ^a^
	SR_180_		188.9 ^a^	45.4 ^a^	13.9 ^a^	88.4 ^b^	21 ^abc^	73.3 ^a^	14.7 ^a^
	SR_225_		181.4 ^ab^	41.2 ^c^	13.4 ^a^	85.7 ^c^	20.2 ^abc^	70.9 ^b^	14.4 ^a^
N_290_	SR_135_		181.7 ^ab^	40.8 ^c^	13.5 ^a^	89.1 ^b^	20.7 ^abc^	69.2 ^c^	14.4 ^a^
	SR_180_		190.4 ^a^	43.5 ^b^	14.1 ^a^	91.2 ^a^	22.5 ^a^	73 ^a^	14.8 ^a^
	SR_225_		184.9 ^a^	38.1 ^d^	13.9 ^a^	89.5 ^ab^	21.7 ^ab^	71.4 ^b^	14.5 ^a^
F-Value	N		845.1 **	2010.4 **	322.7 **	5132.3 **	83.3 **	24379 **	595 **
	SR		19.80 **	36.8 **	4.35 *	19.9 **	2.02	2256 **	3.63 *
	N*SR		0.98	13.9 **	0.2	5.6 *	0.66	172.9 **	0.3

Note: N, SR, and NA represent nitrogen application, seed rate, and N accumulation, respectively. Different letters in the same column represent significant differences in mean values of three replicate plots according to Duncan′s test; * *p* ≤ 0.05 and ** *p* ≤ 0.001, respectively. Revision as above.

**Table 6:** Combination effects of N and SR on N use efficiency parameters in 2018–2019.

N Rate kg ha^−1^	SR kg ha^−1^	NA_E_	NR_E_	NUP_E_	NPFP	NHI
		kg kg^−1^	%	kg kg^−1^	kg kg^−1^	%
N_0_	SR_135_					0.75 ^f^
	SR_180_					0.77 ^de^
	SR_225_					0.77 ^de^
N_180_	SR_135_	9.0 ^f^	39 ^e^	0.9 ^c^	31.2 ^c^	0.76 ^ef^
	SR_180_	10.7 ^de^	44.9 ^cd^	1.02 ^a^	37.3 ^a^	0.78 ^bc^
	SR_225_	10 ^e^	42 ^de^	0.98 ^a^	36.9 ^a^	0.79 ^ab^
N_235_	SR_135_	14.4 ^a^	53.4 ^a^	0.95 ^b^	30.8 ^c^	0.79 ^ab^
	SR_180_	14.4 ^a^	55.2 ^a^	1.02 ^a^	34 ^b^	0.79 ^ab^
	SR_225_	13.5 ^b^	52.7 ^a^	0.98 ^b^	33.3 ^b^	0.8 ^a^
N_290_	SR_135_	13.4 ^b^	48.8 ^b^	0.8 ^d^	27.4 ^e^	0.79 ^ab^
	SR_180_	12.1 ^c^	48.6 ^b^	0.83 ^d^	28.9 ^d^	0.79 ^ab^
	SR_225_	11.4 ^cd^	47.3 ^bc^	0.81 ^d^	28.4 ^de^	0.79 ^ab^
F-value	N	156.4 **	82.6 **	3774.8 **	180.8 **	17.6 **
	SR	6.4 **	4.4 *	20.04 **	55.4 **	7.1 **
	N*SR	8.76 **	2.16	4.27 **	9.12 **	1.88

Note: N, SR, NA_E_, NR_E_, NUP_E_, NPFP, and NHI represent nitrogen rate, seed rate, nitrogen agronomy efficiency, N recovery efficiency, N uptake efficiency, N partial factor productivity, and N harvest index, respectively. Different letters in the same column represent significant differences in mean values of three replicate plots according to Duncan′s test; * *p* ≤ 0.05 and ** *p* ≤ 0.001, respectively.

**Table 7:** Correlation analysis of grain yield with agronomic and photosynthesis traits.

	GY	NS	NGS	TGW	PH	HI	Pn	Gs	SPAD	LAI
**GY**	**1**	0.854 **	0.893 **	0.626 **	0.116	0.879 **	0.917 **	0.788 **	0.889 **	0.961 **
**NS**		1	0.535 **	0.418 *	0.346*	0.733 **	0.641 **	0.492 **	0.638 **	0.777 **
**NGS**			1	0.613 **	−0.08	0.804 **	0.942 **	0.860 **	0.909 **	0.900 **
**TGW**				1	0.212	0.432 **	0.676 **	0.566 **	0.699 **	0.633 **
**PH**					1	−0.102	0.031	−0.138	0.22	0.117
**HI**						1	0.747 **	0.660 **	0.673 **	0.821 **
**Pn**							1	0.852 **	0.951 **	0.923 **
**Gs**								1	0.813 **	0.814 **
**SPAD**									1	0.894 **
**LAI**										1

Note: GY: grain yield; NS: number of spikes; NGS: number of grains per spike; TGW: 1000 grain weight per gram; PH: plant height (cm); HI: harvest index; Pn: net photosynthesis; Gs: stomatal conductance; SPAD: leaf greenness; LAI: leaf area index. * and ** mean significantly correlation at * *p* ≤ 0.05 and ** *p* ≤ 0.001, respectively.

**Table 8:** Equations for estimating dry matter and nitrogen translocation.

Parameters		Equation	Unit
Abbreviation	Denotation		
**PTA**	Pre-anthesis DM translocation	DM of vegetative parts at anthesis—at maturity	kg ha^−1^
**CPT**	Contribution of pre-anthesis DM translocation to grain	PTA ÷ GY at maturity × 100	%
**PAA**	Post-anthesis DM accumulation	Biomass at maturity- biomass at anthesis	kg ha^−1^
**CPA**	Contribution of post-anthesis DM accumulation to grain	PAA ÷ GY at maturity × 100	%
**NTA**	Pre-anthesis N translocation	N of vegetative parts at anthesis—at maturity	kg ha^−1^
**CTG**	Contribution of pre-anthesis N translocation to grain	NTA ÷ grain N×100	%
**NAA**	Post-anthesis N accumulation	Plant N accumulation at maturity—N accumulation at anthesis	kg ha^−1^
**CAG**	Contribution rate of post-anthesis N accumulation to grain	NAA ÷ grain N ×100	%

**Table 9:** Equations for estimating NU_E_ parameters.

Parameters		Equation	Unit
Abbreviation	Denotation		
NA_E_	N agronomy efficiency	(GY with N—GY without N) ÷ N application rate	kg kg^−1^
NR_E_	N recovery efficiency	(total N uptake with N- total N uptake without N) ÷ N application rate	%
NUP_E_	N uptake efficiency	Above-ground N at harvesting ÷ N application rate	%
NPFP	N partial factor productivity	GY ÷ N application rate	kg kg^−1^
NHI	N harvest index	Grain N accumulation at maturity/plant N accumulation at maturity	mg mg^−1^

## Data Availability

The data presented in this study are available on request from the corresponding author.
